# Optimization of a Solvent Exchange Method Enabling the Use of Dehydrated Cellulose Nanofibers as the Thickener in Lubricating Oleogels

**DOI:** 10.3390/gels10110690

**Published:** 2024-10-24

**Authors:** María García-Pérez, Claudia Roman, Samuel D. Fernández-Silva, Miguel A. Delgado, Moisés García-Morales

**Affiliations:** Pro2TecS, Research Center in Chemical Processes and Product Technology, Escuela Técnica Superior de Ingeniería, Universidad de Huelva, Campus “El Carmen”, 21007 Huelva, Spain; maria.gperez@diq.uhu.es (M.G.-P.); samuel.fernandez@diq.uhu.es (S.D.F.-S.); miguel.delgado@diq.uhu.es (M.A.D.); moises.garcia@diq.uhu.es (M.G.-M.)

**Keywords:** cellulose nanofiber, castor oil, oleogel, biogrease, viscoelasticity, solvent exchange

## Abstract

A method that enabled the formulation of lubricating oleogels using dried cellulose nanofibers (CNFs) as an eco-friendly thickener in castor oil was studied. In their dehydrated state, strong hydrogen bonding between nanofibers and high hydrophilicity are the main obstacles to their dispersion in oil. Hence, clusters of dried CNFs had to be previously detached by their dispersion in water. The resulting hydrogels were then subjected to methanol washes to displace the water from the nanofibers. After centrifugation, the methanol-wetted precipitate was readily dispersed in castor oil, forming an oleogel once the methanol was removed. Optimization was conducted in terms of the following variables: (a) hydrogel processing method; (b) hydrogel pH; (c) methanol/hydrogel ratio; (d) number of washes; and (e) oleogel CNF concentration. Their effect on the oleogel linear viscoelastic behavior was analyzed. In general, they demonstrated a prevailing elastic behavior denoted by a well-developed plateau region. The CNF concentration was found to have a more remarkable impact on the oleogels’ rheological behavior than any other variable studied. Hence, substantial differences were observed between 1 and 2 wt.%. The CNFs exhibited a very remarkable thickening capacity in castor oil, achieving a plateau modulus of ca. 700 Pa with just 2 wt.%. Moreover, the resulting oleogels maintained a uniform texture even after one year of storage. This indicates that the oleogels were both homogeneous and storage stable, effectively overcoming the stability issues associated with direct dispersion of dried CNFs in castor oil.

## 1. Introduction

The efforts being made towards a more sustainable and ecological industry result in an increasing demand for eco-friendly alternatives [1,2]. The lubrication industry, traditionally dominated by mineral-oil-based lubricants, resides within this context. In that sense, there is a great concern for the deposition and accumulation of such environmentally unacceptable lubricants in soil and waters [3]. The leading strategy is the introduction of vegetable oils as the base oil, since these are renewable, more biodegradable and present a much lower eco-toxicity [4]. Furthermore, vegetable oils have been proved to possess outstanding lubricity properties [5]. The major limitation of vegetable oils is their low thermal and oxidative stability, requiring practices such as their chemical modification [6] or the use of additives [7] for demanding applications.

Among lubricants, greases also pose the challenge of finding eco-friendly thickeners. Such semi-solid lubricants acquire their consistency and gel-like rheological behavior through a thickening agent that forms a percolated structure that traps the oil [8]. Traditionally, metallic soaps in a 5–30 wt.% concentration are used for this purpose, which compromises the sustainability and biodegradability of the final product [9].

In that regard, cellulose is a natural polymer of great interest, for it is renewable, biodegradable, non-toxic and highly available [10]. The main difficulty that prevents its potential application as a bio-thickener is its low compatibility with base oils. The high polarity and strong intermolecular hydrogen bonds in cellulose contribute to its sedimentation in most oils [11].

Different chemical modifications have been proposed with the aim of obtaining cellulose that can be successfully dispersed [12,13,14,15,16]. However, these methods tend to increase both the environmental impact and the overall cost. Alternatively, nanocellulose has gained attention due to its appealing properties, including a high aspect ratio, large specific surface area, low density and high mechanical strength [17]. Nanocellulose can be produced as cellulose nanofibers (CNFs) or cellulose nanocrystals (CNCs) [17]. Such two types of nanoparticles differ in morphology, size and crystallinity. CNFs appear to be a better candidate for formulating greases due to their morphology and higher aspect ratio. CNFs are fibrillar particles composed of both crystalline and amorphous regions, with diameters between 2 and 60 nm and lengths of several micrometers [18,19]. They form an entangled micro/nanostructure in the lubricant, which is of major relevance for improving the grease performance [20].

The physicochemical properties of CNFs are highly dependent on the nanofibrillation technique used [21,22], which typically involves mechanical processing preceded by pretreatment to reduce energy consumption. The pretreatment relevant for this study is carboxymethylation, a green two-step chemical process. Firstly, activated alkali cellulose is obtained by treating cellulose with sodium hydroxide [23]. Secondly, through a reaction with monochloroacetic acid (esterification reaction), carboxymethyl groups replace three hydroxyl groups in the cellulose molecules [23]. This substitution results in charged COO^−^ groups on the CNF surface, facilitating easier isolation through electric repulsion [24]. Independently of the manufacturing method, CNFs are produced in a gel-like aqueous dispersion, often referred to as hydrogel. However, the presence of water is a significant drawback of these hydrogels, which hinders their direct dispersion in hydrophobic materials [25].

Roman et al. [26] and Ilyin et al. [27] reported methods to successfully disperse CNFs in an oily base for its use as eco-thickener of lubricating greases. Both emphasized the importance of the compatibility of the CNFs with the chosen base oil to ensure stable dispersion. In this context, Ilyin et al. [27] proposed using highly polar oil. The preparation of an oleogel was carried out by high shear dispersion of a microfibrillated cellulose hydrogel in triethyl citrate, followed by water removal in a heat oven. Roman et al. [26] used castor oil, known for its high content of ricinoleic acid, which contains a hydroxyl group that facilitates hydrogen bonding with the CNFs. However, since the binding energy for the CNF–water H-bond is higher than for the CNF–ricinoleic acid H-bond, the authors initially proposed a water–methanol solvent exchange to weaken the CNF–water interaction. This approach was found to be effective, as methanol successfully displaced water molecules away from the cellulose nanofibers. Subsequently, the methanol-wetted CNF was dispersed into the base oil, and any residual methanol was removed under a vacuum. In both methods, the starting material consisted of CNFs in their original production form, i.e., gel-like aqueous suspension, which is essential for their proper use and handling. However, CNFs are usually commercialized as dry powder to facilitate their storage and reduce transportation costs.

Regarding dehydrated CNFs, it should be noted that the drying process leads to partly irreversible agglomeration, such that CNFs can be in bundles that may even reach microscale diameters. Water removal reduces the distance between the fibers, leading to hydrogen bonding between hydroxyl groups on their surface [28]. These intermolecular bonds are very strong, such that a considerable part of the CNFs does not detach, even upon redispersion in water [29]. This phenomenon is known by the term hornification [30,31,32,33,34].

In this manner, the application of dehydrated nanocellulose (both CNFs and CNCs) in the formulation of lubricants has been constrained by poor storage stability due to the fiber propensity to agglomerate and sediment [35,36]. Consequently, such nanoparticles have been used as an additive rather than a primary thickening agent in grease formulations [37]. In view of this, the need to develop methods taking advantage of dried nanocellulose becomes evident. This approach would reduce transportation costs, promoting large-scale application.

As compared to the method by Roman et al. [26], which utilized never-dried nanocellulose, we herein present the use of dehydrated carboxymethylated CNFs as the thickening agent to prepare bio-oleogels based on castor oil. Previously, CNF hydrogels were prepared and used as vehicles to deliver nanofibrillated cellulose into the oil. In this study, with the goal of optimizing the processing protocol, we have dived into the effect of different variables that may affect the rheological properties of the oleogels.

## 2. Results and Discussion

The proposed method of redispersion of dried CNFs in water, followed by solvent exchange with methanol, has been demonstrated to be effective for preparing castor-oil-based oleogels. With the aim of studying the effect of specific processing parameters used during the experimental procedure, nine oleogels were prepared, as outlined in Table 1.

**Figure 1:**
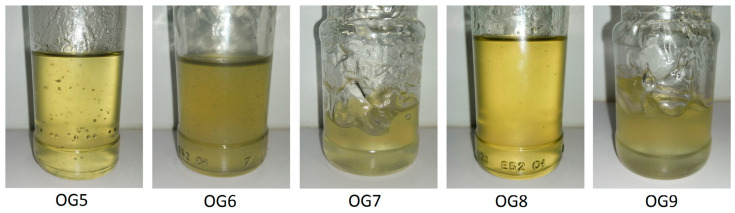
Visual inspection of five selected oleogels: OG5, OG6 and OG9 at 1 wt.% CNFs; and OG7 and OG9 at 2 wt.%. OG6 to OG9, prepared by method (a) are homogeneous; OG5, prepared by method (b), contains solids.

Homogeneous and stable oleogels were successfully obtained, with the exemption of sample OG5, which presented suspended solid particles. This issue will be addressed in detail later. Visual inspection of five selected oleogels is shown in Figure 1. It evidences uniformity in the sample structure with no sign of phase separation. It also reflects a substantial variation in consistency according to their concentrations, 1 and 2 wt.% CNFs. Detailed information about the oleogel formulations is provided in Table 1. In terms of consistency, the 2 wt.% oleogels (OG7 and OG9) achieved a fully gel-like texture, resembling more closely the oleogels reported in the study by Roman et al. [26]. This result confirms the effectiveness of dehydrated CNFs as a potential thickener in oleogel formulations. Within a narrow interval of low concentrations, it can be effectively used to modulate the flow behavior of the oleogels, making them suitable for applications that require tailored viscosity.

The homogeneous aspect observed in Figure 1 is a direct consequence of their excellent stability. Their uniform texture was consistently maintained even after one year, with no changes in physical appearance and no evidence of sedimentation, further supporting their long-term stability. Hence, linear viscoelasticity tests were used for assessing their stability after 6 and 12 months. The results showed no statistically significant differences at a 95 percent confidence level when compared to freshly prepared oleogels.

Optimization of those processing variables which were presumed key to homogeneity, storage stability and rheological performance was conducted by following the sequential study detailed below.

### 2.1. Effect of the Hydrogel Processing Method

**Figure 2:**
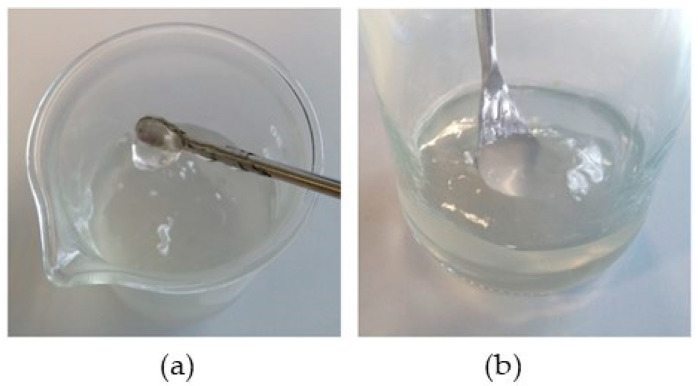
Visual inspection of two selected hydrogels: (**a**) prepared by direct dispersion; (**b**) prepared by dilution–concentration.

All our attempts to disperse highly agglomerated dried CNFs in oil, and even previous trials in methanol, failed. Conversely, their dispersion in water, a solvent with a high dielectric constant, was successfully achieved. According to the supplier, low CNF concentrations may facilitate their dispersion. Under such circumstances, two strategies were proposed for evaluation: (a) direct dispersion of dried CNFs in water at a concentration of 2 wt.%; and (b) dilution–concentration, which consisted in preparing a diluted 1 wt.% hydrogel to enhance nanofiber deagglomeration and dispersion and then, increasing its concentration to 2 wt.% by evaporation. In both methods, it was after the sonication step (Figure 10) that the turbid dispersion of the CNFs became transparent, which indicates that the nanofibrils are effectively dispersed. Although the 1 wt.% dispersions did not present a gelled consistency, they did gel after concentration to 2 wt.%. For the sake of illustrating the effective dispersion of powdered CNFs in water, photographs of two selected 2 wt.% hydrogels are presented. In this respect, Figure 2a corresponds to a hydrogel prepared by method (a), whereas Figure 2b corresponds to a hydrogel prepared by method (b). These images offer a clear visual inspection of the product homogeneity. A priori, no difference was noticed between them. Given the consistency of the fluids, the images also demonstrate that gelling indeed took place using a concentration of 2 wt.% CNFs.

**Figure 3:**
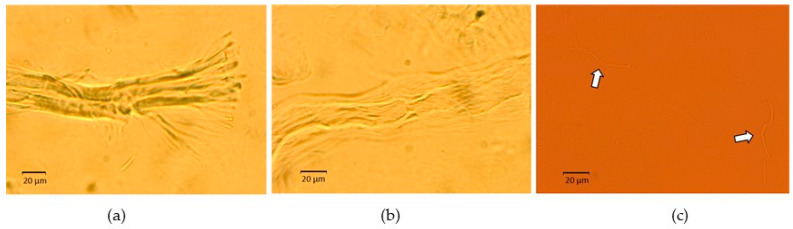
Micrographs of a 2 wt.% hydrogel prepared at pH = 7 using method (**a**). (**a**) Partially disintegrated microfibers with trunk/branch-like Structures, (**b**) Loosely packed clumps of micro/nanofibers, and (**c**) Highly disrupted fibers with significantly reduced diameters.

**Figure 4:**
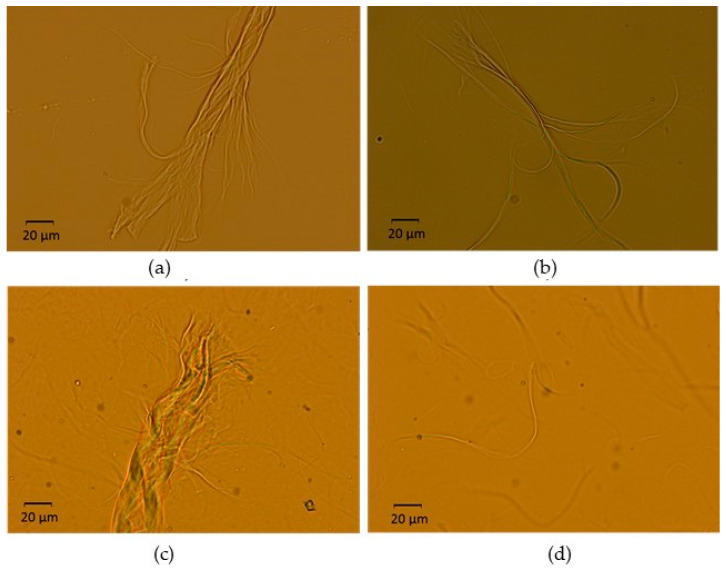
Micrographs of a hydrogel prepared at pH = 7 using method (**b**). (**a**,**b**) before concentration (1 wt.%); (**c**,**d**) after concentration (2 wt.%).

To look further into the distribution of dried CNFs in aqueous medium and assess whether fiber separation occurred, Figure 3, Figure 4 and Figure 6 provide visual inspections of the hydrogels using optical microscopy. These inspections consider a set of experimental variables that are key to the oleogel formation, including pH and different methods of hydrogel preparation.

Figure 3 illustrates a hydrogel prepared by method (a) at 2 wt.% CNFs and pH = 7. As seen in Figure 3a, some microfibers have not been completely disintegrated into nanosized fibers. Even so, water seems to have opened their ends, yielding a trunk/branch-like structure. Such partial swelling suggests that some fibers nearly preserve their initial aggregation. Other microfibers have undergone a more pronounced disruption, resulting in loosely packed clumps of micro/nanofibers (Figure 3b). Nevertheless, fibers with significantly smaller diameters are also present, as depicted in Figure 3c.

On the other hand, Figure 4 shows micrographs of a hydrogel prepared by method (b). Figure 4a,b displays 1 wt.% hydrogel just after sonication, whereas Figure 4c,d presents its final aspect when it was concentrated to 2 wt.%. As in the previous case, agglomerated fibers can be still found, as observed in Figure 4a,c. However, there also seems to be a larger fraction of CNF that has undergone size (diameter) reduction that approaches the nanoscale (Figure 4b,d).

Hence, optical microscopy allowed us to conclude that neither of the two reported methods enabled a 100% exfoliation degree of the microfibers. Similarities were noted between these micrographs and those of reported CNF aqueous dispersions that were directly produced by nanofibrillation, i.e., never-dried nanocellulose [38,39,40]. These dispersions also contain clusters of microfibers that appear more open at their tips, as well as clusters of micro/nanofibers. Yuan et al. [39] demonstrated that increasing the grinding time during nanofibrillation caused fibers to initially form a trunk/branch-like structure. As the grinding continued, the fibers further opened with the breakage of hydrogen bonds between them, which led to thinner fibrils. We hypothesize that a similar deagglomeration process of fibers occurred in our hydrogels during sonication due to the high energy input. This process could have been enhanced at 1 wt.% through increased hydrogen bonding between water molecules and the CNFs. Such improvement in diameter reduction seemed to be maintained after subsequent concentration to 2 wt.% (Figure 4d).

In this sense, the rehydration of commercial CNFs might yield similar results in terms of the final oleogel compared to the use of hydrogels produced at the nanofibrillation step of nanocellulose production. In fact, these may also contain partially fibrillated cellulose. Li et al. [41] developed CNF-based walnut oleogels that exhibited a higher fraction of nanofibers between 5 and 15 nm, but also included occasional fibers of micrometric diameter. These oleogels demonstrated remarkable stability and strength.

**Figure 5:**
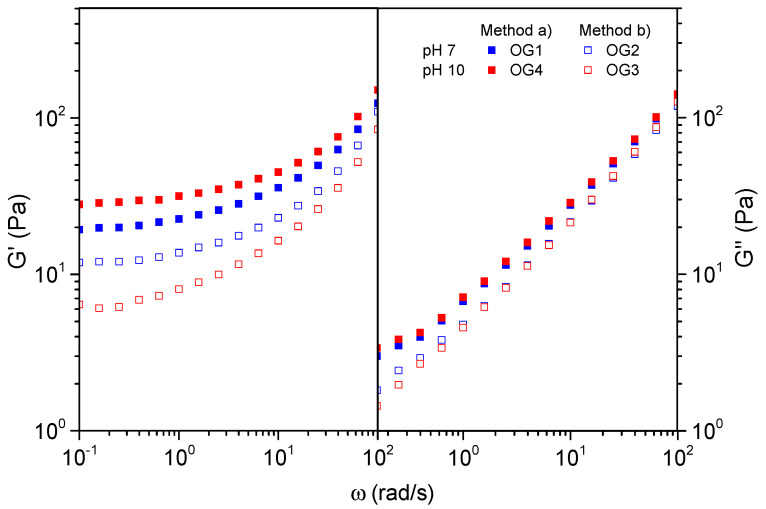
Evolution of the linear elastic and viscous moduli with frequency, at 30 °C, for four selected oleogels prepared by method (a) (OG1, OG4) and (b) (OG2, OG3).

In order to evaluate the influence of the hydrogel preparation on the viscoelastic behavior of the oleogel, two couples of oleogels were selected and compared. The first couple, OG1 and OG2, was produced at pH = 7, whereas the second couple, OG4 and OG3, was produced at pH = 10. Within each couple, the hydrogel preparation method, (a) and (b), respectively, was the only differing variable. Figure 5 displays the dynamic shear frequency sweeps, conducted at stress values in the LVE region.

All the samples showed a prevailing solid-like behavior, denoted by G′ values that were noticeably higher than G″ within the entire frequency interval studied. No matter the pH, the oleogels produced from hydrogels that were prepared by direct dispersion of CNFs, i.e., OG1 and OG4, exhibited a remarkably higher storage modulus (G′) at the low-frequency window. This reveals a stronger network that brings about a more pronounced solid-like behavior compared to the oleogels prepared via dilution–concentration (OG2 and OG3). Considering the previous discussion regarding the quality of hydrogel dispersion, it was anticipated that method (b) (dilution–concentration) would yield oleogels with superior rheological behavior and enhanced stability of the CNF suspension. This is because thinner fibers would promote a more entangled fiber network structure [39,42]. However, an operational difficulty in method (b) compromised the homogeneity of the product. When the 1 wt.% hydrogel was subjected to vacuum rotary evaporation, some gelled lumps formed on the flask wall. The effective oleogel CNF concentration diminished because of the lump formation; hence, a weaker gel behavior was obtained. In some trials, the gelling was so severe that obtaining oleogels with no presence of solids was completely unfeasible. As an example, OG5 is displayed in Figure 1.

In light of this outcome, the direct dispersion of CNFs at 2 wt.%, i.e., method (a), was the only strategy followed in further steps of the optimization protocol. By using method (b), some improvement was found in terms of cellulose exfoliation, as shown in Figure 4b,d. Despite that, method (b) yielded a poorer rheological response and demanded additional time and energy input.

### 2.2. Effect of pH

To further enhance the optimization of direct dispersion of the dried CNFs in water, the alkaline conditions were evaluated. To achieve this, the pH of specific CNF hydrogels was raised from 7 to 10. This adjustment was made based on the carboxymethylated CNFs, which are expected to enhance nanofiber separation through electrostatic repulsion of their resulting anionic carboxylate groups (-COO^−^). More specifically, calculations conducted by Wågberg et al. [43] for carboxymethylated CNFs reflect that the complete dissociation of carboxyl groups into ions occurs within the pH range of 7 to 10, depending on the salt concentration. Our hydrogel, in its unaltered state, exhibited a pH of 7, which serves as the baseline for comparison with the hydrogel adjusted to a pH of 10.

**Figure 6:**
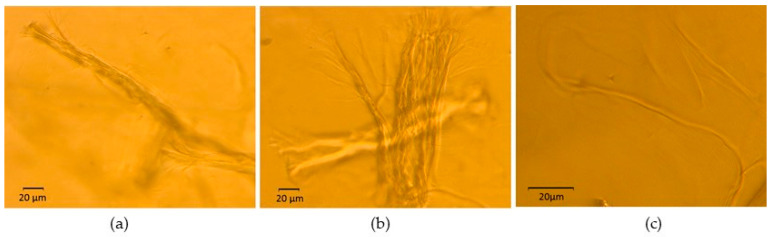
Micrographs of a 2 wt.% hydrogel prepared with method (**a**) at pH = 10. (**a**) non-fibrillated fibers, (**b**) clusters of micro/nanofibers, and (**c**) very fine, nearly disintegrated fibers.

A hydrogel with a pH = 10 was used as a base to formulate different oleogels, specifically, OG3, OG4, OG8 and OG9. To gain a deeper understanding of the potential influence of the hydrogel pH on the fiber dispersion and the degree of fibrillation, optical microscopy was conducted. Figure 6 presents three micrographs that provide a broad view of the fiber arrangement within the hydrogel. Microscopically, the analysis shows that hydrogels with pH = 10 do not lead to notorious differences compared to those with pH = 7 (Figure 3). Both display non-fibrillated fibers (Figure 6a), clusters of micro/nanofibers (Figure 6b) and very fine, nearly disintegrated fibers (Figure 6c).

**Figure 7:**
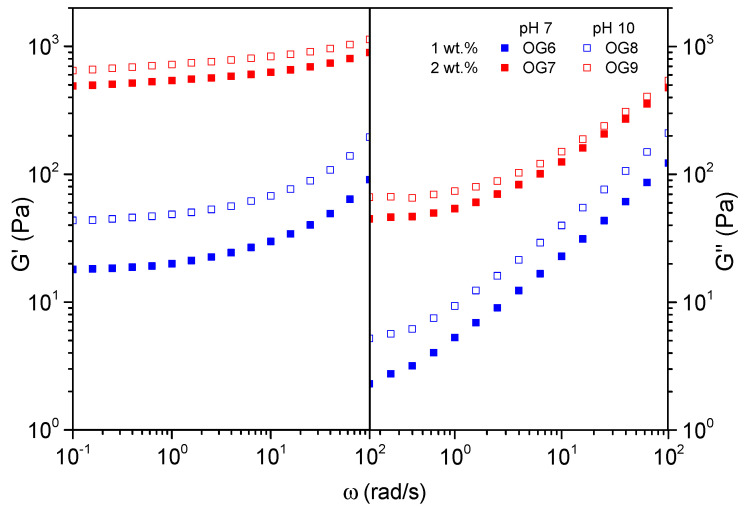
Evolution of the linear elastic and viscous moduli with frequency, at 30 °C, for four selected oleogels prepared at pH = 7 (OG6, OG7) and pH = 10 (OG8, OG9).

To better understand the effect of pH on the oleogel strength, Figure 7 illustrates the evolution of the linear elastic and viscous moduli with frequency for two selected couples of oleogels. The first couple, OG6 and OG8, contained 1 wt.% CNFs, whereas the second couple, OG7 and OG9, contained 2 wt.% CNFs. Within each couple, the pH, 7 and 10, respectively, was the only differing variable.

When oleogels with the same CNF concentration are compared in Figure 7, an increase in both viscoelastic moduli is evident for those samples subjected to pH = 10. Such an effect is more noticeable at the lowest concentration studied, that is, 1 wt.%. We found that at 2 wt.%, the plateau modulus increased from 500 to 700 Pa (ratio = 1.4) when the pH was increased from 7 to 10. Moreover, at 1 wt.%, the plateau modulus was raised from 20 to 40 Pa (ratio = 2) when the pH was increased from 7 to 10. In alkaline aqueous medium, the -COO^−^Na^+^ group of carboxymethylated nanocellulose is in the form of -COO^−^ (deprotonated). Electrostatic repulsion among anions occurs. Such a positive effect on the oleogel viscoelastic response at pH = 10 might be a consequence of an improved colloidal stability. In relative terms, the effect is more noticeable at 1 wt.% due most probably to a greater mobility capacity (less topological constraints) of the fibers under the electrostatic repulsions.

In this sense, three potential mechanisms that may have contributed to the gel structuring are identified: (i) physical entanglement of the CNFs [44]; (ii) electrostatic repulsion among the CNFs provided by the newly formed carboxylate anionic groups [43,45]; and (iii) hydrogen bonding among the CNFs and between the CNFs and castor oil through the hydroxyl groups of cellulose and the hydroxyl group present in ricinoleic acid [46]. At a lower concentration of 1 wt.%, the entanglement of CNFs may play a less dominant role in gel formation, thereby allowing electrostatic repulsion to have a greater impact.

In Figure 5, the enhancement due to pH adjustment is also observed when OG1 (pH = 7) and OG4 (pH = 10) are compared. Conversely, the opposite trend is observed for samples OG2 (pH = 7) and OG3 (pH = 10). However, it is important to consider that OG2 and OG3 were obtained from hydrogels produced by method (b). Thus, the positive effect induced by the pH might have been masked by the concentration issues (material loss) previously reported.

**Figure 8:**
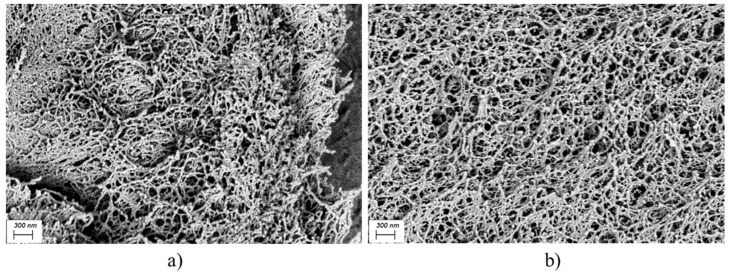
SEM images for two selected 2 wt.% oleogels. (**a**) OG7, prepared at pH = 7; (**b**) OG9, prepared at pH = 10.

The SEM images in Figure 8, corresponding to two selected 2 wt.% oleogels, may provide valuable insights into the issue. Figure 8a,b display oleogels prepared at pH = 7 and pH = 10, respectively. These images demonstrate the positive effect of alkaline pH, yielding an entangled cellulose thickener phase that remains more structurally stable due to -COO^−^ groups exerting electrostatic repulsion among the fibers. It seems that fibers stressed by electrostatic repulsion yield a more porous network.

Both nanofiber networks shown in Figure 8 might be compared with the SEM image reported by Eyholzer et al. [47], showing an aqueous dispersion of dried carboxymethylated CNFs. Attention must be paid to the difference in concentration, as the dispersion presented in the referenced study contains 0.1 wt.% CNFs, whereas our oleogels contain 2 wt.%. This higher concentration accounts for a denser skeleton, with narrower space between the nanofibers. Despite this density, the fibers in Figure 8 are notably finer than those of Eyholzer et al. [47]. This underscores the efficiency of the redispersion process employed in our study, as well as the effective transfer of CNFs to castor oil, maintaining a significantly small CNF diameter. This superior exfoliation of fibers could be attributed to the use of sonication during hydrogel preparation, a more powerful method compared to the blender used by the other authors. In addition to such enhanced diameter reduction, a more homogeneous fiber dispersion can be induced by adjusting the pH to 10, as seen in Figure 8b.

In conclusion, increasing the pH to 10 does not seem to have facilitated nanofiber separation in terms of cross-section reduction. Optical microscopy revealed virtually no changes in the CNFs’ diameter of the hydrogel due to pH modification. However, in castor oil, the contribution of the CNF thickener is enhanced at pH = 10, as supported by the results from rheological tests. Alkaline pH seems to have led to a more cohesive CNF network due to repulsive interactions.

### 2.3. Effect of the Methanol/Hydrogel Ratio

As previously explained, methanol enables water removal from the fibers. Hence, methanol-wetted CNFs can be readily transferred into the oil phase, yielding homogeneous and stable oleogels when the solvent is vacuum-distilled. Following the solvent-exchange method by Roman et al. [26], a first experiment using a methanol/hydrogel ratio of 3/1 was attempted. However, solid–liquid separation was incomplete, even when the centrifugation time was increased from 15 min to 1 h. The supernatant remained very turbid, i.e., with a high content of CNFs. In view of this experience, the methanol/hydrogel ratio had to be increased in the first wash. A 5/1 ratio led to complete separation of the phases in 15 min. In subsequent washes, when most of the water retained by the CNFs had already been removed, a 3/1 ratio was enough. Consequently, the whole set of oleogels, OG1 to OG9, was prepared in the same manner.

### 2.4. Effect of the Number of Washes

**Figure 9:**
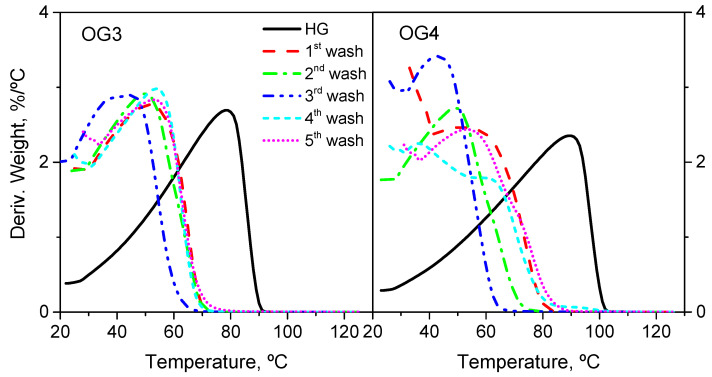
DTG curve for the hydrogels of samples OG3 and OG4, as a function of wash number.

After each wash, water removal was monitored using thermogravimetric analysis (TGA) of the precipitate. Oleogels from OG1 to OG4 were prepared using five washes. TGA measurements confirmed that fourth and fifth washes were, in fact, unnecessary. Figure 9 shows the evolution of the sample weight % first derivative with the temperature for two selected oleogels, OG3 and OG4. Both were subjected to five washes. The thermograms evidenced that water was fully removed after just three washes, rendering the last two steps redundant.

As observed, the solvent peak shifted to a lower temperature as water (normal boiling point of 100 °C) was displaced by methanol (normal boiling point of 64.7 °C). In the fourth and fifth washes, the curve shifted back to a higher temperature. Based on this, we hypothesize that by the third wash, all the free water would have been removed, and that by the fourth wash, the high excess of methanol used would have also replaced the water bound to the fibers. Bound water is water that is directly attached to the surface of the fibers through stronger interactions than free water [31]. This would explain why from the fourth wash onwards, the methanol peak shifted to the right, indicating the presence of bound, rather than free, methanol.

It is worth emphasizing that bound water does not interfere with the compatibilization of CNFs in castor oil and, therefore, does not hinder the formation of a stable oleogel. This was confirmed for oleogels OG6 to OG9, for which only three washes were carried out and the fibers were successfully integrated into the oil. It was found that with less than three washes, the nanocellulose ended up forming lumps. Moreover, four or more washes did not lead to any significant difference in either the dispersion easiness or rheological response with respect to three washes. This was the criterium used.

### 2.5. Effect of the Oleogel CNF Concentration

Lastly, once the previous four processing variables were optimized, the CNF concentration was studied. On these grounds, OG6 and OG7, at pH = 7, and OG8 and OG9, at pH = 10, are compared in Figure 7. In any case, at low frequency, the storage modulus remained almost frequency-independent and consistently greater than the loss modulus, indicating a prevailing elastic response. It is important to stress that the presence of this plateau region is related to the existence of entanglements within the thickener. Hence, all the analyzed oleogels reflect a well-established network structure. However, the results indicate a very noticeable difference in the viscoelastic behavior between oleogels containing 1 wt.% and those with 2 wt.% CNFs. The latter exhibited markedly higher G′ values, with a plateau region that extended to higher frequencies, as well as a minimum in G″. This rheological pattern clearly denotes a stronger gel network and improved elastic properties compared to those with 1 wt.% CNFs [48,49]. 

This finding emphasizes the prospective use of CNFs as a suited thickener for lubricating applications. This offers the potential to achieve significantly different thickening degrees within a small range of low concentrations. Consequently, biogreases with different NLGI grades (different consistencies) could be developed, foreseeably, at much lower concentrations than those used in conventional lubricating greases. Furthermore, different CNF concentrations would yield thickener structures with varying capabilities to trap the base oil, thereby affecting their overall stability and textural properties. Obviously, further studies on the matter (tribology and oil bleeding tests) would be required.

## 3. Conclusions

Eco-friendly oleogels using dried nanocellulose as a thickener were successfully obtained by applying a variant of the method outlined by Roman et al. [26]. In the present study, the procedure started with the preparation of 2 wt.% CNF hydrogels from commercial raw material. This approach effectively addressed the dispersibility drawback that resulted from the direct addition of dried CNF powder in castor oil. The method yielded homogeneous and very stable oleogels with remarkable elastic features. Their linear viscoelastic behavior was enhanced at pH 10. This suggests a more favorable conformation of the nanofibers due to the electrostatic repulsion between carboxylate anions on the CNFs’ surface. Hence, increasing the pH has been identified as key to oleogel preparation when carboxymethylated nanocellulose is involved. As far as the solvent exchange method is concerned, free water needs to be completely removed before the CNFs can be used. Conversely, bound water does not restrain oleogel formation. From a rheological perspective, the substantial difference observed within a narrow range of low concentrations is very noteworthy, proving the outstanding potential of CNFs as a thickening agent. This has a remarkable technical implication in terms of prospective formulation of biogreases with varying NLGI grades. Furthermore, from a practical standpoint, the capacity to obtain the oleogels starting from dehydrated CNFs implies an advantageous reduction in transportation costs as compared to the use of cellulose hydrogels. Finally, it is important to highlight the need for testing a wider variety of dried nanocellulose before drawing further conclusions.

## 4. Materials and Methods

### 4.1. Materials

The CNFs were supplied by NANOGRAFI (Ankara, Turkey) in the form of white dry powder with ca. 4 wt.% moisture. They were produced from cotton cellulose, which is subjected to carboxymethylation. Thus, the surface of the CNFs contained, in addition to hydroxyl groups, carboxyl groups (carboxyl content of 2.5 mmol/g). The specifications for particle size were 10–20 nm wide and 2–3 µm in length. Their crystallinity measured by XRD is 92%, and their density is 1.50 g/cm^3^.

Castor oil supplied by Guinama (Valencia, Spain) was used as the base oil for the oleogels. At 40 °C, it has a kinematic viscosity of 242.5 cSt and a density of 0.948 g/mL. Its fatty acid composition (wt.%) is as follows: palmitic (C16:0): 1.70; stearic (C18:0): 1.96; oleic (C18:1): 5.34; ricinoleic (C18:1:OH): 82.48; linoleic (C18:2): 7.01; linolenic (C18:3): 1.51.

### 4.2. Oleogel Preparation

Nine oleogels were prepared by modifying different variables in the procedure. The solvent-exchange method reported by Roman et al. [26] was taken as reference, with the particularity that in the present study, the CNFs were available as dried nanocellulose instead of in a gel-like aqueous suspension. Therefore, a previous phase of preparation of a hydrogel (HG) by the redispersion of dried CNFs in water was necessary.

**Figure 10:**
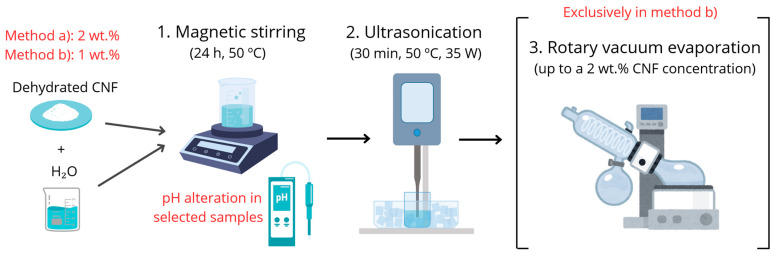
Schematic of the processing protocol of CNF hydrogels.

For the sake of contrasting the quality of dispersion, two routes for the preparation of a 2 wt.% hydrogel were explored: (a) direct preparation: dispersion of CNFs at 2 wt.% in water; and (b) dilution–concentration: initial dispersion of CNFs at 1 wt.% in water, followed by rotary vacuum evaporation of the hydrogel up to a final concentration of 2 wt.% CNFs. In both routes, the CNFs were pre-dispersed in 50 g batches of water using magnetic stirring (50 °C, 24 h). In specific samples, the pH was modified by adding drops of NaOH aqueous solution. This way, the pH was increased from around 7 to 10, which was measured with a Horiba LAQUA PC110 pH-meter. Subsequently, the samples were sonicated using a Hielscher Ultrasonics UP400St ultrasonicator (50 °C, 30 min, 35 W). In route (b), the final step was the concentration of the 1 wt.% hydrogel using a Heidolph Laborota 4001 Rotary Evaporator until a concentration of 2 wt.% was reached. Thus, concentration to half the initial weight was conducted for 30 min, according to a previous calibration test, under the same conditions of 60 °C and 15 rpm. The final concentration was then verified by measuring the weight of the resulting hydrogel. For a comprehensive overview, Figure 10 illustrates the processing protocol of CNF hydrogels, with particular emphasis on the variables studied (preparation method and pH).

The next step for the oleogel preparation was a series of methanol washes, following the protocol defined by Roman et al. [26]. In every wash, fresh methanol was added to the hydrogel. The mixture was then homogenized using a T25 digital Ultra-Turrax homogenizer (10 min, 10k rpm) to induce the solvent-exchange by high-shear dispersion. Different methanol/hydrogel ratios were studied. Following that, the separation of a methanol-wetted nanocellulose precipitate from a water–methanol supernatant was achieved using a Thermo Scientific™ Sorvall™ ST 8 Small Benchtop Centrifuge (15 min, 4.5k rpm). This sequence was repeated for several washes aiming at the complete displacement of water from the CNFs, which was monitored by TGA measurements. The number of washes was also a variable of study.

In the last step, the methanol-wetted CNFs were manually dispersed in the weight of castor oil required to obtain oleogels of the desired concentration (the options studied were 1 and 2 wt.%). After dispersing for 10 min, the methanol was then removed from the mixture by evaporation under vacuum (60 °C, 60 min, 60 rpm). The ultimate step is the high shear homogenization of the oleogel in an Ultra-Turrax T 25 at 3k rpm for 5 min.

To provide a clear overview of the variables utilized for each sample in this study, Table 1 details the experimental parameters involved in the processing of all nine oleogels.

**Table 1:** Summary of the experimental variables for the processing of each oleogel (OG).

OG Code	HG Preparation Route	HG pH	Methanol/HG Ratio vol/wt	No. Washes	OG Concentration (wt.%)
OG1	(a)	7	5/1 (1st wash), 3/1 (next)	5	1
OG2	(b)	7	5/1 (1st wash), 3/1 (next)	5	1
OG3	(b)	10	5/1 (1st wash), 3/1 (next)	5	1
OG4	(a)	10	5/1 (1st wash), 3/1 (next)	5	1
OG5	(b)	7	5/1 (1st wash), 3/1 (next)	3	1
OG6	(a)	7	5/1 (1st wash), 3/1 (next)	3	1
OG7	(a)	7	5/1 (1st wash), 3/1 (next)	3	2
OG8	(a)	10	5/1 (1st wash), 3/1 (next)	3	1
OG9	(a)	10	5/1 (1st wash), 3/1 (next)	3	2

### 4.3. Hydrogel Characterization

The hydrogels were observed in an Olympus BX51 optical microscope to evaluate the dispersion of the nanofibers. This technique was employed to assess the degree of nanofibrillation of the CNFs in the hydrogel, focusing on fiber dispersion, uniformity and structural integrity. Besides that, to monitor water removal from the samples after the washes, approximately 15 mg precipitate in each wash was subjected to thermogravimetric analysis (TGA) in a Q-50 analyzer (TA Instruments, New Castle, DE, USA). In such tests, the samples were heated from room temperature (approximately 20 °C) to 130 °C at a rate of 10 °C/min, in an inert N2 atmosphere (flow of 50 mL/min).

### 4.4. Oleogel Characterization

The oleogel linear viscoelastic behavior was analyzed by small amplitude oscillatory tests using the controlled stress rheometer Physica MCR 301 (Anton Paar, Gaz, Austria). The test configuration was a parallel plate–plate geometry of 50 mm diameter, with a 1 mm gap between the plates and a constant temperature of 30 °C. Firstly, a strain sweep from 0.03% to 10% was carried out at a frequency of 1 Hz (6.28 rad/s) to determine the region of linear viscoelasticity (LVE), where the viscoelastic parameters are strain-independent. Afterwards, a dynamic shear frequency sweep from 0.1 to 100 rad/s was conducted at a central strain value of the previously measured linear viscoelastic zone to evaluate the linear viscoelastic behavior through the elastic (G′) and viscous (G″) moduli. At least two replicates of these tests were performed for each oleogel.

Morphological observations of the oleogel microstructures were conducted at room temperature with a scanning electron microscope (SEM), model ZEISS EVO LS15 (ZEISS, Oberkochen, Germany), at 10 kV. Previously, all the samples were chemically fixed on the holder using the protocol described elsewhere [26]. The representative morphology prototypes were assured by using at least three different samples and taking five pictures at different locations for each formulation studied.

## Data Availability

Data will be made available on request.

## References

[1] UN Department of Economic and Social Affair (2023). The Sustainable Development Goals Report 2023: Special Edition.

[2] Prieto-Sandoval V., Mejía-Villa A., Ormazabal M., Jaca C. (2020). Challenges for ecolabeling growth: Lessons from the EU Ecolabel in Spain. Int. J. Life Cycle Asses..

[3] Shah R., Woydt M., Zhang S. (2021). The Economic and Environmental Significance of Sustainable Lubricants. Lubricants.

[4] Mannekote J.K., Kailas S.V., Venkatesh K., Kathyayini N. (2018). Environmentally friendly functional fluids from renewable and sustainable sources-A review. Renew. Sustain. Energy Rev..

[5] Adhvaryu A., Erhan S.Z., Perez J.M. (2004). Tribological studies of thermally and chemically modified vegetable oils for use as environmentally friendly lubricants. Wear.

[6] Lasch G., Stradolini P., Gehlen G.S., Barros L.Y., Polleto J.C., Ramalho A., Fernandes C.M.C.G., Romio P.C., Petzhold C.L., Ferreira N.F. (2024). Comparative tribological investigation of castor oil and its transesterified and aminolyzed derivatives. Tribol. Int..

[7] Quinchia L.A., Delgado M.A., Franco J.M., Spikes H.A., Gallegos C. (2012). Low-temperature flow behaviour of vegetable oil-based lubricants. Ind. Crop. Prod..

[8] Delgado M.A., Valencia C., Sánchez M.C., Franco J.M., Gallegos C. (2006). Influence of Soap Concentration and Oil Viscosity on the Rheology and Microstructure of Lubricating Greases. Ind. Eng. Chem. Res..

[9] Tian C., Xu H., Dong J. (2003). A novel layered double hydroxides oleogel lubricant: Inspired by conventional greases. Tribol. Int..

[10] Wei H., Rodriguez K., Renneckar S., Vikesland P.J. (2014). Environmental science and engineering applications of nanocellulose-based nanocomposites. Environ. Sci. Nano.

[11] Chu Y., Sun Y., Wu W., Xiao H. (2020). Dispersion Properties of Nanocellulose: A Review. Carbohydr. Polym..

[12] Gallego R., Cidade T., Sánchez R., Valencia C., Franco J.M. (2016). Tribological behaviour of novel chemically modified biopolymer-thickened lubricating greases investigated in a steel–steel rotating ball-on-three plates tribology cell. Tribol. Int..

[13] Sánchez R., Franco J.M., Delgado M.A., Valencia C., Gallegos C. (2011). Gel-like dispersions based on cellulosic derivatives and castor oil applicable as biodegradable lubricating greases. Chem. Eng. Trans..

[14] Sánchez R., Franco J.M., Delgado M.A., Valencia C., Gallegos C. (2011). Thermal and mechanical characterization of cellulosic derivatives-based oleogels potentially applicable as bio-lubricating greases: Influence of ethyl cellulose molecular weight. Carbohydr. Polym..

[15] Martín-Alfonso J.E., Núñez N., Valencia C., Franco J.M., Díaz M.J. (2011). Formulation of new biodegradable lubricating greases using ethylated cellulose pulp as thickener agent. J. Ind. Eng. Chem..

[16] Cortés-Triviño E., Valencia C., Delgado M.A., Franco J.M. (2018). Rheology of epoxidized cellulose pulp gel-like dispersions in castor oil: Influence of epoxidation degree and the epoxide chemical structure. Carbohydr. Polym..

[17] Xie H., Du H., Yang X., Si C. (2018). Recent Strategies in Preparation of Cellulose Nanocrystals and Cellulose Nanofibrils Derived from Raw Cellulose Materials. Int. J. Polym. Sci..

[18] Tayeb A.H., Amini E., Ghasemi S., Tajvidi M. (2018). Cellulose Nanomaterials—Binding Properties and Applications: A Review. Molecules.

[19] Xu X., Liu F., Jiang L., Zhu J.Y., Haagenson D., Wiesenborn D.P. (2013). Cellulose Nanocrystals vs. Cellulose Nanofibrils: A Comparative Study on Their Microstructures and Effects as Polymer Reinforcing Agents. ACS Appl. Mater. Interfaces.

[20] Zhou C., Ren G., Fan X., Lv Y. (2022). Probing the effect of thickener microstructure on rheological and tribological properties of grease. J. Ind. Eng. Chem..

[21] Lee S.-Y., Chun S.-J., Kang I.-A., Park J.-Y. (2009). Preparation of cellulose nanofibrils by high-pressure homogenizer and cellulose-based composite films. J. Ind. Eng. Chem..

[22] Patil T.V., Patel D.K., Dutta S.D., Ganguly K., Santra T.S., Lim K.-T. (2022). Nanocellulose, a versatile platform: From the delivery of active molecules to tissue engineering applications. Bioact. Mater..

[23] Kanikireddy V., Varaprasad K., Jayaramudu T., Karthikeyan C., Sadiku R. (2020). Carboxymethyl cellulose-based materials for infection control and wound healing: A review. Int. J. Biol. Macromol..

[24] Lavoine N., Desloges I., Dufresne A., Bras J. (2012). Microfibrillated cellulose—Its barrier properties and applications in cellulosic materials: A review. Carbohydr. Polym..

[25] Rajinipriya M., Nagalakshmaiah M., Robert M., Elkoun S. (2018). Importance of Agricultural and Industrial Waste in the Field of Nanocellulose and Recent Industrial Developments of Wood Based Nanocellulose: A Review. ACS Sustain. Chem. Eng..

[26] Roman C., García-Morales M., Eugenio M.E., Ibarra D., Martín-Sampedro R., Delgado M.A. (2021). A sustainable methanol-based solvent exchange method to produce nanocellulose-based ecofriendly lubricants. J. Clean. Prod..

[27] Ilyin S.O., Gorbacheva S.N., Yadykova A.Y. (2023). Rheology and tribology of nanocellulose-based biodegradable greases: Wear and friction protection mechanisms of cellulose microfibrils. Tribol. Int..

[28] Xu Y., Xu Y., Chen H., Gao M., Yue X., Ni Y. (2022). Redispersion of dried plant nanocellulose: A review. Carbohydr. Polym..

[29] Ding Q., Zeng J., Wang B., Tang D., Chen K., Gao W. (2019). Effect of nanocellulose fiber hornification on water fraction characteristics and hydroxyl accessibility during dehydration. Carbohydr. Polym..

[30] Posada P., Velásquez-Cock J., Gómez-Hoyos C., Serpa A.M., Lyulin S.V., Kenny J.M., Gañán P., Castro C., Zuluaga R. (2020). Drying and redispersion of plant cellulose nanofibers for industrial applications: A review. Cellulose.

[31] Peng Y., Gardner D.J., Han Y. (2012). Drying cellulose nanofibrils: In search of a suitable method. Cellulose.

[32] Sellman F.A., Benselfelt T., Larsson P.T., Wågberg L. (2023). Hornification of cellulose-rich materials—A kinetically trapped state. Carbohydr. Polym..

[33] Kwak H.W., You J., Lee M.E., Jin H.-J. (2019). Prevention of cellulose nanofibril agglomeration during dehydration and enhancement of redispersibility by hydrophilic gelatin. Cellulose.

[34] Missoum K., Bras J., Belgacem M.N. (2012). Water Redispersible Dried Nanofibrillated Cellulose by Adding Sodium Chloride. Biomacromolecules.

[35] Delgado-Canto M.A., Fernández-Silva S.D., Roman C., García-Morales M. (2020). On the Electro-Active Control of Nanocellulose-Based Functional Biolubricants. ACS Appl. Mater. Interfaces.

[36] Awang N.W., Ramasamy D., Kadirgama K., Samykano M., Najafi G., Sidik N.A.C. (2019). An experimental study on characterization and properties of nano lubricant containing Cellulose Nanocrystal (CNC). Int. J. Heat Mass Tran..

[37] Li J., Ling N., Du C., Ge Y., Amann T., Feng H., Yuan C., Li K. (2022). Tribological behavior of cellulose nanocrystal as an eco-friendly additive in lithium-based greases. Carbohydr. Polym..

[38] Fonseca A.d.S., Panthapulakkal S., Konar S.K., Sain M., Bufalinof L., Raabe J., Miranda I.P.d.A., Martins M.A., Tonoli G.H.D. (2019). Improving cellulose nanofibrillation of non-wood fiber using alkaline and bleaching pre-treatments. Ind. Crop. Prod..

[39] Yuan T., Zeng J., Wang B., Cheng Z., Chen K. (2021). Cellulosic Fiber: Mechanical Fibrillation-Morphology-Rheology Relationships. Cellulose.

[40] Wang W., Mozuch M.D., Sabo R.C., Kersten P., Zhu J.Y., Jin Y. (2016). Endoglucanase Post-Milling Treatment for Producing Cellulose Nanofibers from Bleached Eucalyptus Fibers by a Supermasscolloider. Cellulose.

[41] Li X., Guo G., Zou Y., Luo J., Sheng J., Tian Y., Li J. (2023). Development and Characterization of Walnut Oleogels Structured by Cellulose Nanofiber. Food Hydrocol..

[42] Zou Y., Tian Y., Zhao B., Li J., Luo J., Sheng J., Li X. (2024). Effects of Cellulose Diameter on the Formation and Rheological Properties of Edible Walnut Oleogels Structured by Cellulose Nanofiber. Food Hydrocol..

[43] Wågberg L., Decher G., Norgren M., Lindström T., Ankerfors M., Axnäs K. (2008). The Build-Up of Polyelectrolyte Multilayers of Microfibrillated Cellulose and Cationic Polyelectrolytes. Langmuir.

[44] Alves L., Magalhães S., Pedrosa J.F.S., Ferreira P.J.T., Gamelas J.A.F., Rasteiro M.G. (2024). Rheology of Suspensions of TEMPO-Oxidised and Cationic Cellulose Nanofibrils—The Effect of Chemical Pre-Treatment. Gels.

[45] Mendoza L., Batchelor W., Tabor R.F., Garnier G. (2018). Gelation Mechanism of Cellulose Nanofibre Gels: A Colloids and Interfacial Perspective. J. Colloid Interface Sci..

[46] Roman C., Delgado M.A., Fernández-Silva S.D., García-Morales M. (2022). Exploring the Effect of the Pulp Bleaching on the Thermo-Rheological Behavior of Sustainable Cellulose Nanofiber-Based Oleogels. J. Environ. Chem. Eng..

[47] Eyholzer C., Bordeanu N., Lopez-Suevos F., Rentsch D., Zimmermann T., Oksman K. (2010). Preparation and Characterization of Water-Redispersible Nanofibrillated Cellulose in Powder Form. Cellulose.

[48] Ross-Murphy S.B. (1994). Rheological characterization of polymer gels and networks. Polym. Gels Netw..

[49] Ross-Murphy S.B. (1995). Structure–property relationships in food biopolymer gels and solution. J. Rheol..

